# Theoretical insights into the structural, optoelectronic, thermoelectric, and thermodynamic behavior of novel quaternary LiZrCoX (X = Ge, Sn) compounds based on first-principles study

**DOI:** 10.1039/d3ra03815g

**Published:** 2023-10-10

**Authors:** Meena Kumari, Jisha Annie Abraham, Ramesh Sharma, Debidatta Behera, S. K. Mukherjee, Mostafa M. Salah, Murefah mana Al-Anazy, Mohammed S. Alqahtani

**Affiliations:** a Department of Physics, National Defence Academy Pune 411023 India disisjisha@yahoo.com; b Department of Applied Physics, Defence Institute of Advanced Technology Girinagar Pune-411025 India; c Dept. of Applied Science, Feroze Gandhi Institute of Engineering and Technology Raebareli Uttar Pradesh India sharmadft@gmail.com; d Dept. of Physics, Birla Institute of Technology Mesra Jharkhand-835215 India; e Electrical Engineering Department, Future University in Egypt Cairo 11835 Egypt mostafa.abdulkhalek@fue.edu.eg; f Department of Chemistry, College of Sciences, Princess Nourah bint Abdulrahman University (PNU) P.O. Box 84428 Riyadh 11671 Saudi Arabia; g Department of Radiological Sciences, College of Applied Medical Sciences, King Khalid University Abha 61421 Saudi Arabia

## Abstract

The structural, magnetic, electronic, elastic, vibrational, optical, thermodynamic as well as thermoelectric properties of newly predicted quaternary LiZrCoX (X = Ge, Sn) Heusler compounds are evaluated intricately with the aid of *ab initio* techniques developed under the framework of density functional theory. The computed structural properties are found to be in tandem with the existing analogous theoretical and experimental facts. Structural optimization has been carried out in three different structural arrangements, *i.e.*, Type-1, Type-2, and Type-3. Further analysis of the optimization curves reveals that the Type-3 phase, which has the least amount of energy, is the most stable structure for the compounds under consideration. The tabulated cohesive energy and formation energy of these compounds depict their chemical as well as thermodynamic stability. The absence of negative phonon frequencies in the phonon band spectrum of the studied compounds depicts their dynamic stability. Similarly, the tabulated second-order elastic constants (*C*_*ij*_) and the linked elastic moduli show their stability in the cubic phase. The calculated value of Pugh's ratio and Cauchy pressure reveal that LiZrCoGe is brittle whereas LiZrCoSn is ductile. Additionally, the optical characteristics of the compounds are studied in terms of the dielectric function, refractive index, extinction coefficient, absorption coefficient, reflectivity, energy loss function, and optical conductivity. The obtained high value of power factor and figure of merit of the studied lithium-based quaternary compounds predict good thermoelectric behavior in these compounds. Thus, LiZrCoX (X = Ge, Sn) compounds can therefore be used to create innovative and intriguing thermoelectric materials as well as optoelectronic and energy-harvesting equipment.

## Abstract 2

Quaternary LiZrCoX (X = Ge, Sn) Heusler compounds are assessed for their structural, magnetic, electrical, elastic, vibrational, optical, thermodynamic, and thermoelectric properties. The strong power factor and figure of merit values demonstrate good thermoelectric behaviour.

## Graphical abstract

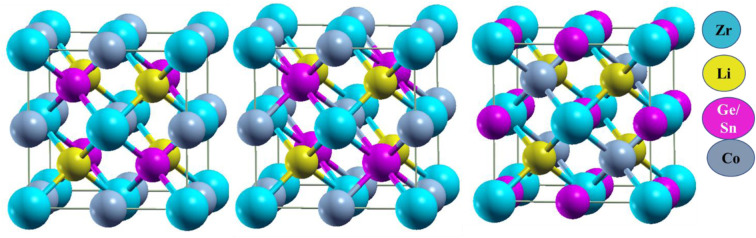

## 1. Introduction

The successful completion of spacecraft missions in the last 20 years has attracted great attention towards thermoelectric generators using thermoelectric alloys that provide long-term power.^1^ The energy conversion efficiency of this thermoelectric is determined by the figure of merit (*ZT*) using the relation *ZT* = *S*^2^*σT*/(*κ*_l_ + *κ*_e_), where *S*, *σ*, *T*, and (*κ*_l_ + *κ*_e_) are the Seebeck coefficient, electrical conductivity, absolute temperature, and lattice and electronic contribution towards the thermal conductivity of the solids, respectively.^2–4^ The power factor (*S*^2^*σ*) or figure of merit (*ZT*) can be increased by diminishing the value of the thermal conductivity of the solid and increasing electrical conductivity. Low thermal conductivity *κ*_l_ can be accomplished by isoelectronic alloying, defect engineering, *etc.*^5–7^ With the advent of science and advances, it is obligatory to design operative thermoelectric alloys with augmented electronic, elastic, and mechanical properties. The discovery of a novel class of Heusler compounds has made it possible to realize such prospects for the advancement of modern technology. Heusler compounds are mainly of three categories, *i.e.*, ternary Heusler alloys (ABC), full Heusler alloys (A_2_BC), and quaternary Heusler alloys EQH (AA′BC).^8^ When one of the A atoms in the full Heusler alloys is replaced with another type of atom A′, EQH alloys are obtained. EQH alloys possess less disorder in comparison to the ternary Heusler.^9^ The EQH alloys, however, are created by filling any suitable voids between half Heuslers or full Heuslers, which comply with the 18-Valence Electron Count (VEC) rule, using an appropriate electropositive element, such as Li. They can be easily tuned for designing various electronic as well as transport properties for varied applications in optoelectronics, thermoelectric devices, *etc.* Several theoretical as well as experimental studies have been conducted recently to gain insight into the electronic behavior as well as the thermoelectric behavior of these systems.^10–14^ First-principles calculations were utilised by Haleoot *et al.* (2020) to analyse the exceptional thermoelectric and thermodynamic properties of the Quaternary Heusler (QH) compounds CoFeYGe (Y = Ti, Cr).^15^ Density functional theory (DFT) has been used to examine another novel quaternary Heusler compound, CrVNbZn, which has the space group 216 (of cubic geometry) and shows maximum *ZT* = 0.79 in the wide temperature range of 260 K to 480 K as well as excellent electronic and magnetic properties.^16^ Recently, the structural, electronic, and magnetic properties of the equiatomic quaternary Heusler alloy ZnCdRhMn using the first-principles calculations and Monte Carlo simulations have been investigated by Idrissi *et al.*^17^ Recently, first principles-based calculations have been performed for Li-based quaternary compounds LiHfCoX (Ge, Sn) by Kaur *et al.*^14^ Abraham *et al.* have conducted a comparative analysis of various exchange–correlation functionals aimed at predicting the structural, electrical, optical, and transport features of the new quaternary LiTiCoSn.^18^ Gupta *et al.* have investigated the ground state characteristics of a semiconducting new quaternary Heusler alloy LiScPdPb.^19^ Singh *et al.* have investigated the vibrational, thermoelectric transport, and mechanical properties of the lithium-based Heusler compound LiTiCoSn using a plane wave pseudopotential approach.^20^ Structural, electronic, mechanical, and thermoelectric properties of LiTiCoX (X = Si, Ge) compounds have also been investigated by Singh *et al.*^21^ Some of the quaternary Heusler compounds based on Li that have recently undergone productive examination^39,40^ for their structural, electrical, and thermoelectric properties are LiScPdPb, LiTiCoSi, and LiTiCoGe. Because of the high melting point as well as complying with the typical 18 valence electron counts, the Li based EQH has the capacity to design high-efficiency thermoelectric devices. In light of these motives, in this work, we have constrained ourselves to the study of lithium-based EQH LiZrCoX (X = Ge, Sn) alloys. The comparatively smaller atomic size of the lithium (Li) atom is very apt for filling the vacant space in the Heusler alloy. The single valence electron provided by Li, 4 valence electrons from Zr and Ge/Sn, and nine electrons from Co form a 18 valence electron quaternary Heusler system. As per the literature survey, neither experimental nor theoretical investigations have been conducted on these compounds, which motivated us to initiate studies on them. Using first-principles calculations along with BoltzTraP and PHONOPY, the structural, electrical, mechanical, optical, vibrational, and transport features of these novel compounds LiZrCoX (X = Ge, Sn) are investigated. Section 2 in the current paper describes the calculation procedure, and Section 3 discusses the findings. Finally, Section 4 brings everything together with the conclusions.

## 2. Computational details

The full potential linear augmented plane wave (FP-LAPW) method^22^ and the DFT framework have been used to determine the physical characteristics of LiZrCoX (X = Ge, Sn) quaternary Heuslers. The generalized gradient approximation (GGA) is used to predict the structural, elastic, and electronic properties in the WIEN2k code.^23,24^ Birch–Murnaghan equation of state is used to determine structural parameters by fitting the energy *vs.* volume curve. The *R*_MT_*K*_max_ value is chosen to be 8, and the number of *k*-points in the first Brillouin zone is the 15 × 15 × 15 mesh according to the Monkhorst–Pack scheme.^25^ The valence wave functions are expanded to *l*_max_ = 10 partial waves, inside the atomic spheres. Up to *G*_max_ = 12 a.u^−1^, the potential and charge density are expanded. The IRELAST method,^26^ implemented in the WIEN2k package, is used to compute the elastic constants. The Seebeck coefficient (*S*), electrical, and thermal conductivities, as well as other thermoelectric properties of these materials as a function of temperature are also computed using the semiclassical BoltzTraP algorithm.^27^ The rigid band approximation and constant relaxation time are the fundamental ideas behind this code. We also determine the cubic elastic mechanical stability using the relaxed structure derived using the PBE-GGA approach. We were able to get thermodynamic parameters such as the Debye temperature by solving the Gibbs function within the quasi-harmonic Debye model using the Gibbs2 code.^28^ The quasi-harmonic Debye model for LiZrCoX (X = Ge, Sn) is used in the Gibbs program to examine the thermal effects over the temperature range of 0 to 1200 K. Additionally, the VASP software is used to generate the cubic LiZrCoX phonon spectrum (X = Ge, Sn) using the pseudopotential plane-wave method.^29^ The computation uses a 450 eV plane-wave cutoff. For the computations, an 18 × 18 × 18 *k*-mesh that is a denser mesh is employed for the IBZ integration of the unit cell.

## 3. Results and discussion

### 3.1. Structural properties

**Fig. 1:**
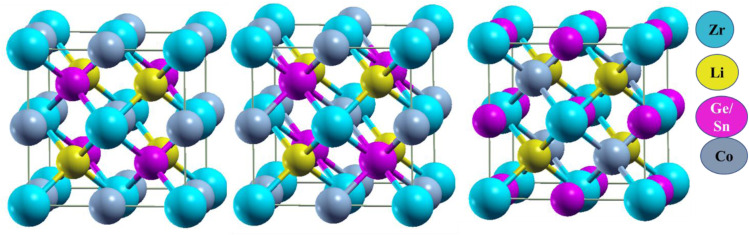
Crystal structure of LiZrCoX (X = Ge, Sn).

**Fig. 2:**
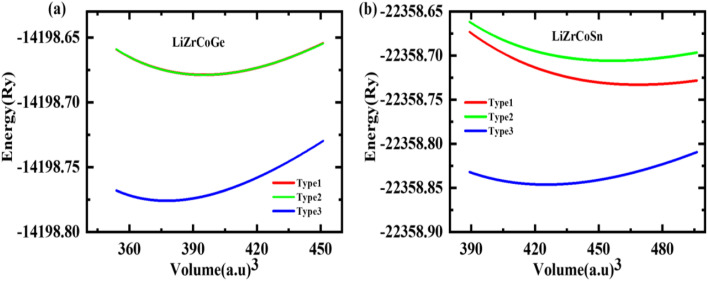
The energy *vs.* volume diagram for LiZrCoX (X = Ge, Sn).

The investigated quaternary Heusler compounds LiZrCoX (X = Ge, Sn) crystallize in cubic phase with space group *F*43*m* (SG#216), as shown in Fig. 1.^30,31^ There are three possible arrangements of atomic sites, namely Type-1, Type-2, and Type-3. The Wyckoff positions corresponding to these three arrangements are tabulated in Table 1.^32^ The volume optimization of these compounds has been performed in these three arrangements, and the variation of total energy is plotted against volume for both the investigated compounds. The energy *vs.* volume curves of LiZrCoX (X = Ge, Sn) are displayed in Fig. 2(a) and (b). The energy *vs.* volume plot for LiZrCoGe overlaps in Type-1 and Type-2 phases. It can be deduced from Fig. 2 that both the investigated systems crystallize in Type-3 arrangement as the energy–volume curve lies below the other two arrangements. The optimized lattice parameters along with equilibrium energy and bulk modulus and its pressure derivative of the investigated LiZrCoX (X = Ge, Sn) are presented in Table 2. From the equilibrium energy values presented in Table 2, it can be inferred that the studied compounds have minimum energy in the Type-3 phase.^33^

**Table 1:** Atomic positions with the crystal structure of XX′YZ alloy at different crystal sites resulting in three different types (where X = Li, X′ = Ti, Y = Co, and Z = Sn)

Type	Atom			
	Li	Zr	Co	Ge/Sn
Type-1	D (0.75, 0.75, 0.75)	B (0.25, 0.25, 0.25)	C (0, 0, 0)	A (0.5, 0.5, 0.5)
Type-2	D (0.75, 0.75, 0.75)	C (0, 0, 0)	B (0.5, 0.5, 0.5)	A (0.25, 0.25, 0.25)
Type-3	D (0.75, 0.75, 0.75)	A (0, 0, 0)	C (0.25, 0.25, 0.25)	B (0.5,0.5,0.5)

**Table 2:** Calculated lattice parameter *a* (Å), volume (Å^3^), optimized minimum energy (*E*_min_), bulk modulus (*B*) GPa, pressure derivative (*B*′) of XX′YZ alloy (where X = Li, X′ = Ti, Y = Co, and Z = Ge/Sn)

Compound		*a* (Å)	*V* (Å)^3^	*B* (GPa)	*B*′	*E*_min_ (Ry)
LiZrCoGe	Type 1	6.27	416.46	101.64	3.72	−14 198.688473
	Type 2	6.16	394.66	113.69	4.32	−14 198.677175
	Type 3	6.07	378.33	134.42	4.65	−14 198.775025
LiZrCoSn	Type 1	6.52	468.73	94.32	4.26	−22 358.732856
	Type 2	6.46	456.29	93.07	5.45	−22 358.705692
	Type 3	6.31	425.30	117.76	3.84	−22 358.846198

All of our subsequent research has been based on a Type-3 setup, with PBE-GGA serving as the exchange–correlation potential and Birch–Murnaghan equation of state being used to match the optimized energy *vs.* volume data.^34^ The obtained data of ground state properties are tabulated in Table 3. The optimized lattice parameters, unit-cell volume, equilibrium energy, and bulk modulus and its pressure derivative along with bond length, formation energy, and cohesive energy are presented in Table 3. The lattice parameter of LiZrCoSn is found to be larger than that of LiZrCoGe, which might be due to the increase in the size of Sn than Ge. The bulk modulus of LiZrCoGe is greater than LiZrCoSn using PBE-GGA exchange correlation potential; hence, LiZrCoGe is found to be stiffer than LiZrCoSn. The bond length of both the studied LiZrCoX (X = Ge, Sn) has also been computed and presented in Table 3. The bulk modulus of the investigated LiZrCoX (X = Ge, Sn) decreases as we go from Ge to Sn due to their elongated bond lengths. The thermodynamic and chemical stabilities of the explored quaternary Heusler LiZrCoX (X = Ge, Sn) alloys have been evaluated by calculating their cohesive energy and formation energy, which is illustrated in Table 3. Cohesive energy can be used for quantifying the atomic binding stability of compounds. The calculation for the cohesive energy per atom is as follows:1
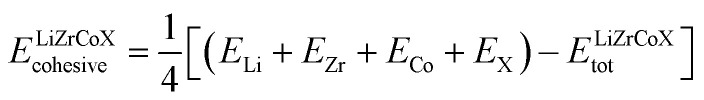
where *E*_LiZrCoX_ is the total energy of the unit cell in the cubic phase and *E*_Li_, *E*_Zr_, *E*_Co_, and *E*_X_ are the energies of individual atoms. The tabulated value of cohesive energy of LiZrCoGe and LiZrCoSn is 4.84 eV per atom and 4.61 eV per atom, respectively, indicating their chemical stability due to stronger chemical bonds among the constituent atoms.^35–37^

**Table 3:** Calculated lattice parameters (*a* in Å), unit-cell volume (*V*_0_, in Å^3^), equilibrium energy (*E*_min_ in Ry), bulk modulus (*B* in GPa) and its pressure derivative *B*′, bond length (Å), formation energy (Δ*H*, in kJ mol^−1^) and cohesive energy (*E*_coh_, in eV per atom)

Compounds	Parameters	LiZrCoGe	LiZrCoSn	Another study^14^
PBE-GGA	*a* (Å)	6.07	6.31	6.05
	*V* (Å)^3^	378.33	425.30	
	*B* (GPa)	134.42	117.76	131
	*B*′	4.65	3.84	
	*E*_min_ (Ry)	−14 198.775025	−22 358.846198	
	Band gap (eV)	1.142	0.971	1.45
Bond length (Å)		Ge–Zr = 3.03	Sn–Zr = 3.15	
		Ge–Co = 2.63	Sn–Co = 2.73	
		Zr–Li = 2.63	Zr–Li = 2.73	
		Zr–Co = 2.63	Zr–Co = 2.73	
Formation energy	Δ*H* (eV per atom)	−0.539	−0.520	
Cohesive energy	*E*_cohesive_ (eV per atom)	4.84	4.61	
Effective mass	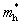	0.40	0.36	
	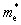	0.85	0.92	

The formation energy per atom is evaluated with the help of the following relation:2
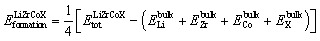
*E*^LiZrCoX^_tot_ is the total energy of LiZrCoX (X = Ge, Sn) per formula unit, and *E*^bulk^_Li_, *E*^bulk^_Zr_, *E*^bulk^_Co_, and *E*^bulk^_X_ are the total energies of Li, Zr, Co, and Ge/Sn bulk, respectively. It is observed from Table 3 that the investigated compounds have negative formation energy, demonstrating that their thermodynamic stability and formation process involves exothermic phenomenon. The evaluated formation energy per atom and cohesive energy per atom of LiZrCoX (X = Ge, Sn) shown in Table 3 reveal their stability in the cubic phase.

**Fig. 3:**
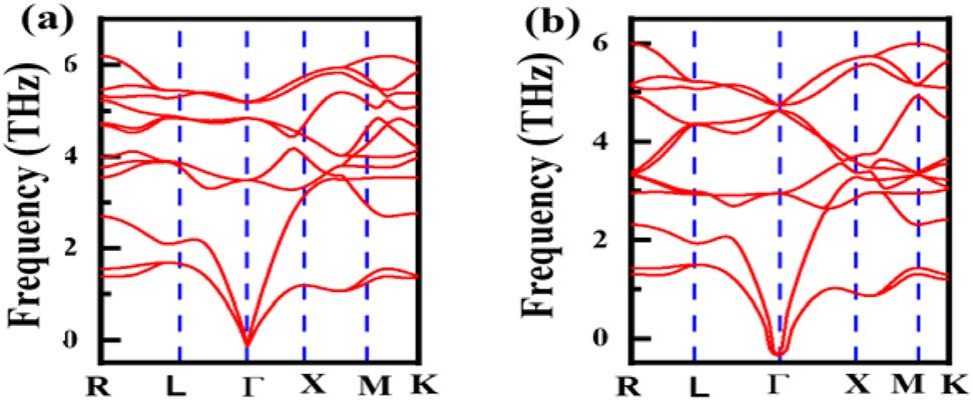
Phonon dispersion plot for LiZrCoX (X = Ge, Sn).

Phonon dispersion (PD) against momentum is computed to verify the stability of the LiZrCoX (X = Ge, Sn), as shown in Fig. 3(a) and (b). The modes in the phonon dispersion plot generally look like spaghetti with transverse and longitudinal modes. We used momentum along the *x*-axis and frequency (THz) along the *y*-axis in the range of 0 to 6 THz. We discovered a small amount of negative frequency in the LiZrCoX (X = Ge, Sn) compounds, although almost all modes are positive and have actual phonon branches. According to the literature, LiZrCoX (X = Ge, Sn) will be stable if modest pressure levels (GPa) are applied.^38,39^

### 3.2. Magnetic properties

The valence electronic configuration of the investigated Li–Zr quaternary alloys LiZrCoX (X = Ge, Sn) includes Li[2s^1^], Zr[5s^2^4d^2^], Co[3d^7^4s^2^], Ge[4s^2^4p^2^], Sn[5s^2^5p^2^], forming 18 valence electron count alloys. The presence of the ferromagnetic element cobalt (Co) in LiZrCoX (X = Ge, Sn) motivated us to perform spin-polarized calculations to determine the magnetic moment of the compound.^40–42^ The result of spin polarized computations reveals that the total magnetic moment of both the investigated compounds are found to be zero, which agrees well with the Slater–Pauling rule (*M*_T_ = *Z*_T_ − 18) *μ*_B_, in which *M*_T_ denotes the total magnetic moment, *Z*_T_ is the count of total valence electrons in the compound, and *μ*_B_ is Bohr magneton.^43,44^ Therefore, our further investigation of compounds has been carried out in the non-magnetic phase.

### 3.3. Electronic properties

**Fig. 4:**
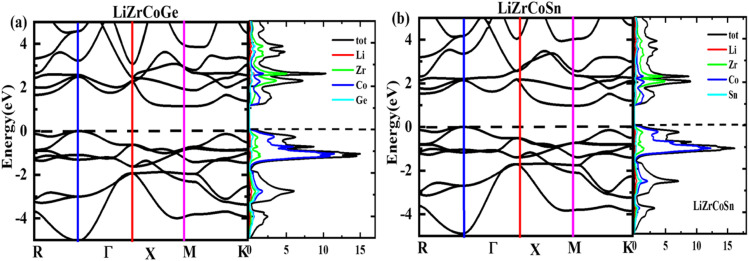
Band structure and density of states plot for (a) LiZrCoGe and (b) LiZrCoSn.

**Fig. 5:**
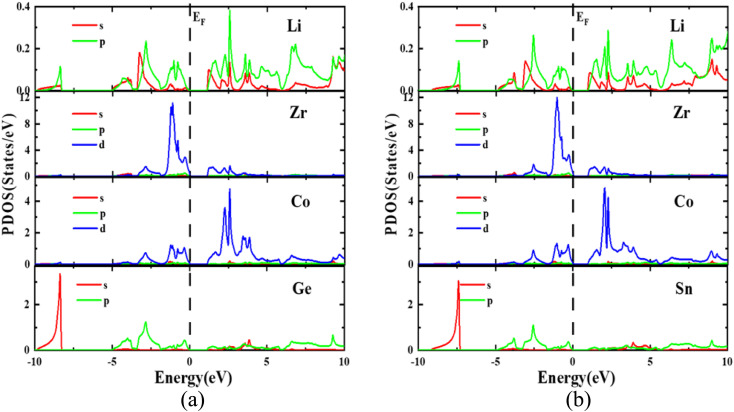
Partial density of states for (a) LiZrCoGe and (b) LiZrCoSn.

The optimized lattice parameters obtained using PBE-GGA are used to elucidate the electronic properties using band structure as well as density of states plots for the investigated LiZrCoX (X = Ge, Sn). The electronic band structures and density of states are plotted along the high symmetry principal directions along *R*–*Γ*–*X*–*M*–*K* and presented in Fig. 4(a), (b) and 5(a), (b). It is evident from Fig. 4(a) and (b) that both the explored quaternary Heusler alloys possess an indirect band gap of 1.142 eV (LiZrCoGe) and 0.971 eV (LiZrCoSn) between their valence band and conduction band. It is observed from the band plots that the Fermi level *E*_F_, which is set to 0 eV, lies near the valence band in both compounds, depicting its p-type semiconductor nature.^45^ The valence band near the Fermi level is formed due to the ‘d’-like states of Co and Zr and ‘p’-like states of Ge/Sn, in which the main contribution belongs to the Zr atom in both compounds. The lowest lying band lies in between −10.0 eV and −9.0 eV (−10.0 eV and −7.0 eV), as observed in Fig. 5(a) and (b), due to the ‘s’-like states of Ge (Sn). As seen in Fig. 5(a) and (b), the conduction band near the Fermi level arises due to the hybridization of Zr, Co, and Ge/Sn, in which the dominant contribution belongs to the ‘d’-like states of Co. The conduction bands near the Fermi level are shifted towards *E*_F_, resulting in a reduction in the energy gap, while moving from LiZrCoGe to LiZrCoSn. Even though the contribution from the lithium atom towards the electronic behavior of these compounds is negligible, as observed from the band plots given in Fig. 4 and 5, it provides one valence electron to the other atoms, giving a semiconducting nature. The computed effective masses for LiZrCoX along the high symmetry principal directions of the Brillouin zone are tabulated in Table 3.

Studying the effective masses of carriers (holes and electrons) is crucial for improving the understanding of photovoltaic properties, which are highly influenced by resistivity, carrier mobility, and optical response of free carriers. In LiZrCoX (X = Ge, Sn), the effective mass of carriers is calculated using the following formula.3
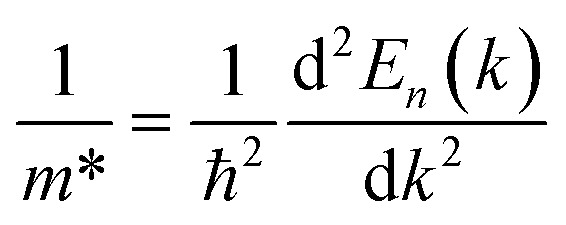


The estimated effective mass values for both the investigated LiZrCoX compounds are provided in Table 3. The findings demonstrate that the examined quaternary half Heusler alloys have very small effective masses of carriers (electron and hole). The decreased effective mass is highly beneficial for solar materials since it makes carrier transfer easier. Accordingly, LiZrCoX (X = Ge, Sn) may be effective in photovoltaic applications.

### 3.4. Elastic and mechanical properties

Both the investigated LiZrCoX (X = Ge, Sn) crystallize in the cubic phase; therefore, they possess three independent second-order elastic constants (SOECs), namely, *C*_11_, *C*_12_, and *C*_44_. The calculated SOECs of LiZrCoX using PBE-GGA are tabulated in Table 4. The computed SOECs obey Born–Huang stability criteria^46,47^ given by

4 *C*_11_ − *C*_12_ > 0; *C*_11_ > 0; *C*_44_ > 0, *C*_11_ + 2*C*_12_ > 0, *C*_12_ < *B* < *C*_11_

which reveals the mechanical stability of the investigated LiZrCoX (X = Ge, Sn) compounds in the cubic phase under ambient conditions. These elastic constants help us to evaluate the mechanical durability, elasticity, and strength to defend the deformation forces on the studied compounds. *C*_44_ refers to the resistance to stress-oriented deformations and *C*_11_ quantifies the stiffness of the materials against applied stress on them.^48^ The computed shear modulus as well as Young's modulus are higher for LiZrCoSn than that of LiZrCoGe, predicting LiZrCoSn to be stiffer than LiZrCoGe. Table 4 presents the computed values of the other related elastic constants, such as shear modus (*G*), Young's modulus (*Y*), Poisson's ratio, and anisotropic factor (*A*). Zener anisotropy factor *A* can be computed using *A* = 2*C*_44_/(*C*_11_ − *C*_12_), and the obtained values for both compounds show deviation from unity, depicting their anisotropic behavior.^49^ The ionic/covalent and ductile/brittle nature of the studied quaternary LiZrCoX (X = Ge, Sn) Heusler compounds can be assessed using certain criteria like Poisson's ratio, Pugh's ratio, and Cauchy pressure. Ductile compounds generally yield Poisson's ratio *σ* > 0.33.^50^ The value of *σ* for central forces in solids and ionic crystals can vary between 0.25 and 0.5,^50^ whereas for covalent materials, the value of *σ* varies between 0.1 and 0.25, and interatomic forces belong to non-central forces.^51,52^ It can be deduced from Table 4 that the computed value of *σ* is found to be 0.23 and 0.26, respectively, for LiZrCoGe and LiZrCoSn, revealing that LiZrCoGe belongs to ionic and LiZrCoSn belongs to covalent materials. According to Pugh's criterion,^53,54^ the material has a ductile nature if its Pugh's ratio is greater than 1.75 and *vice versa*. The calculated Pugh's ratio of LiZrCoGe is 1.58 and that of LiZrCoSn is 1.77, as given in Table 4, confirming the brittle nature of LiZrCoGe and the ductile nature of LiZrCoSn. The brittleness of LiZrCoGe is confirmed by the negative value of Cauchy pressure (*C*_12_ − *C*_44_), and the ductility of LiZrCoSn is also affirmed by the positive value of Cauchy pressure.^55,56^ Besides these, we have also computed longitudinal sound velocity, transverse sound velocity, and average sound velocity of LiZrCoX (X = Ge, Sn). Using these sound velocities, we have calculated Debye temperature (*θ*_D_), a crucial physical entity, which relates various physical properties such as specific heat capacity, thermal conductivity, and melting point of the crystal with elastic constants. At low temperatures, Debye temperature *θ*_D_ can be evaluated from the SOECs. The Debye temperature *θ*_D_ is found to be lower for LiZrCoGe than LiZrCoSn, revealing the possibility of its low lattice thermal conductivity as the Debye temperature is directly related to lattice thermal conductivity.^57^ The melting points corresponding to these explored LiZrCoX compounds are also evaluated using *T*_melt_ = [553 K + (5.911 K GPa^−1^)*C*_11_] ± 300 K and tabulated in Table 4.

**Table 4:** Values of elastic constants (*C*_*ij*_), bulk modulus (*B*), shear modulus (*G*), Young's modulus (*Y*), Poisson's ratio (*σ*), Pugh's ratio, Frantsevich's ratio, Shear anisotropy factor (*A*), Cauchy pressure *C*^P^, sound velocities (m s^−1^), Debye temperature *θ*_D_ (K) of LiZrCoX (X = Ge, Sn)

Material property	LiZrCoGe	LiZrCoSn	Another study^9^
*C*_11_ (GPa)	208.31	310.27	154.9
*C*_12_ (GPa)	89.51	128.53	61.5
*C*_44_ (GPa)	96.14	116.61	62.7
Shear modulus, *G* (GPa)	81.44	106.31	55.72
Young's modulus, *Y* (GPa)	201.88	168.61	
Poisson's ratio, *σ*	0.23	0.26	
Pugh's ratio (GPa)	1.58	1.77	
Shear anisotropy factor, *A*	1.61	1.28	
Cauchy pressure, *C*^P^	−6.63	11.92	
Transverse sound velocity (m s^−1^)	3387	3747	
Longitudinal sound velocity (m s^−1^)	5830	6624	
Average sound velocity (m s^−1^)	3758	4167	
Debye temperature (*θ*_D_)	466.5	550.5	
Melting temperature, *T*_m_	2576.2	4215.6	

### 3.5. Optical properties

**Fig. 6:**
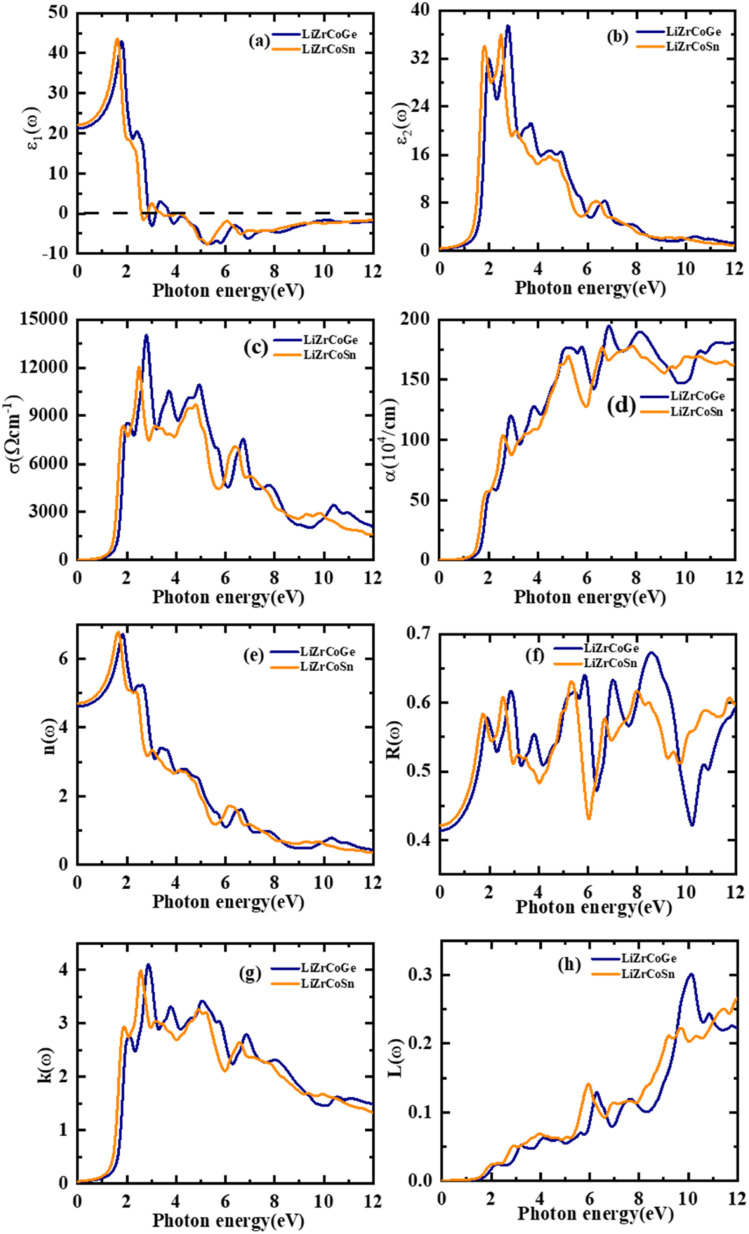
Computed optical spectra (a) real *ε*_1_(*ω*), (b) *ε*_2_(*ω*), (c) conductivity *σ*(*ω*), (d) absorption *α*(*ω*), (e) refractive index *n*(*ω*), (f) reflectivity *R*(*ω*), (g) extinction coefficient *k*(*ω*), and (h) loss function *L*(*ω*) for LiZrCoX (X = Ge, Sn).

The optical characteristics of both the investigated quaternary Heusler compounds LiZrCoX (X = Ge, Sn) are computed and presented in Fig. 6(a)–(h), and the values are tabulated in Table 5. The optical properties are essential for determining the optical performance of the devices, especially for optoelectronics and solar cell applications. The optical properties of these compounds such as refractive index *n*(*ω*), extinction coefficient *k*(*ω*), optical conductivity *σ*(*ω*), and absorption coefficient *α*(*ω*) are determined using the real and imaginary part of the complex dielectric function *ε*(*ω*) = *ε*_1_(*ω*) + i*ε*_2_(*ω*).^58–60^ The dielectric function *ε*(*ω*) depicts how electromagnetic radiation interacts with a given material and how the material responds to it. The value of *ε*_1_(*ω*) and *ε*_2_(*ω*) can be evaluated using the following relations:5
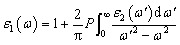
6
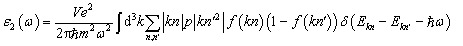
in which *V* is the unit cell volume, *m* is the mass of electron, *e* is its electronic charge, |*kn* is the crystal wave function, *p* is the momentum operator, *f*(*kn*) is the Fermi–Dirac distribution function, and ℏ*ω* is the incident photon energy.

**Table 5:** Optical properties of LiZrCoX (X = Ge, Sn)

Compounds	*ε*_1_(0)	*n*(0)	*R*(0)
LiZrCoGe	20.97	4.60	0.41
LiZrCoSn	22.12	4.70	0.42
Other study^79^	12.32	3.51	

The optical functions such as the electron loss function *L*(*ω*) and *n*(*ω*) can be determined using Kramers–Kronig relations.^61,62^7
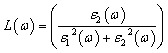
8
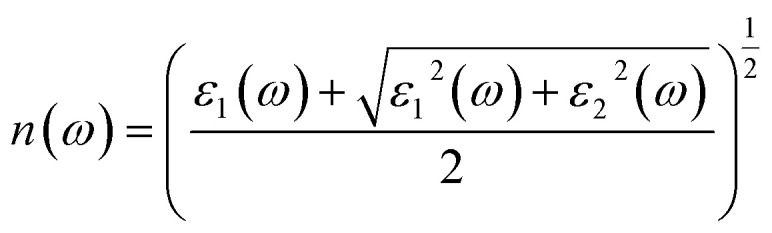


The computed photon spectral dependency of the real as well imaginary part of the complex dielectric function *ε*(*ω*) is consolidated in Fig. 6(a) and (b) for both the investigated compounds LiZrCoX (X = Ge, Sn). The real part *ε*_1_(*ω*) is deduced from the imaginary part *ε*_2_(*ω*), and *ε*_2_(*ω*) represents the absorption behavior of the material.^63^ The static dielectric function *ε*_1_(0) yields the value of 20.97 and 22.12 for LiZrCoGe and LiZrCoSn, respectively. Other reports are in accordance with the computed values for LiZrCOX (X = Ge, Sn). It gives information regarding the electronic polarizability of the materials. The main peak of *ε*_1_(*ω*), as in Fig. 4(a) of LiZrCoGe, is 2.0 eV, which gets shifted to 1.7 eV for LiZrCoSn. After the optimal peak, *ε*(*ω*) diminishes gradually and reaches 0 for LiZrCoGe at 3.0 eV and LiZrCoSn at 2.5 eV. It remains in the negative region up to 12 eV for both the investigated quaternary compounds. The spectral dependency of the imaginary part *ε*_2_(*ω*) with the incident electromagnetic radiation up to 12 eV for both compounds is displayed in Fig. 4(b). The first peak of *ε*_2_(*ω*) of LiZrCoSn occurs at almost 2.0 eV for both compounds whereas the optimal peak occurs at about 3.0 eV for both Li–Zr compounds. *ε*_2_(*ω*) decreases gradually with the increase in energy of incident electromagnetic radiation even though minor peaks occur at 4 eV, 5 eV, and 7 eV.

The spectral dependency of the optical conductivity *σ*(*ω*) of the studied LiZrCoX compounds has been investigated and presented in Fig. 6(c). The optimal peak of optical conductivity occurs at 2.3 eV and 3.0 eV for LiZrCoSn and LiZrCoGe, respectively. Several minor peaks of *σ*(*ω*) occur at various points of the incident photon energy of electromagnetic radiation between 3.0 eV and 12.0 eV.^64^ The incident photon spectral dependency of the absorption spectrum *α*(*ω*) has been computed and illustrated in Fig. 6(d). It displays the optimal peak in the high energy region of the electromagnetic spectrum, depicting them as prominent UV absorbing materials.^63^

The refractive index *n*(*ω*) profiles of the studied compounds are illustrated in Fig. 6(e). The static refractive index value *n*(0) is 4.60 and 4.70 for LiZrCoGe and LiZrCoSn, respectively, and the same is tabulated in Table 5. Other reports are consistent with the LiZrCoX (X = Ge, Sn) calculated values. It increases gradually with incident electromagnetic energy and reaches an optimal value of almost 6.8 around 2 eV for both the investigated compounds and thereafter decreases with the increase in energy of incident electromagnetic radiations. The refractive index profile follows a similar trend as that of the real part of the dielectric function for both explored materials.

The reflectivity spectra *R*(*ω*) with incident electromagnetic radiations for LiZrCoX have been also investigated and presented in Fig. 6(f), which reveals optimal reflection at about 5.8 eV for LiZrCoSn and a shift towards the high energy region, *i.e.*, to 8.3 eV in the case of LiZrCoGe. The extinction coefficient *k*(*ω*) has also been computed for the studied compounds up to incident energy of 12 eV and demonstrated in Fig. 6(g). It is found to have a threshold value below their band gaps and thereafter gradually increases and reaches an optimal value of almost 4 at about 3 eV for both LiZrCoGe and LiZrCoSn,^65^ after which it continuously decreases in the high energy region of the electromagnetic spectrum and follows a similar trend to that of *ε*_2_(*ω*).

The energy loss function *L*(*ω*) has also been computed in the incident photon energy ranging from 0 to 12 eV and is presented in Fig. 6(h). It describes the amount of energy being lost while a fast-traveling electron traverses through the compound. The maximum energy loss function *L*(*ω*) has been found to have a value of 0.3 at 10 eV for LiZrCoGe and it gets shifted towards the high energy region for LiZrCoSn. Zero energy loss is observed in the band gap region with a plodding rise of incident photon energy for both these materials.

### 3.6. Thermodynamic properties

**Fig. 7:**
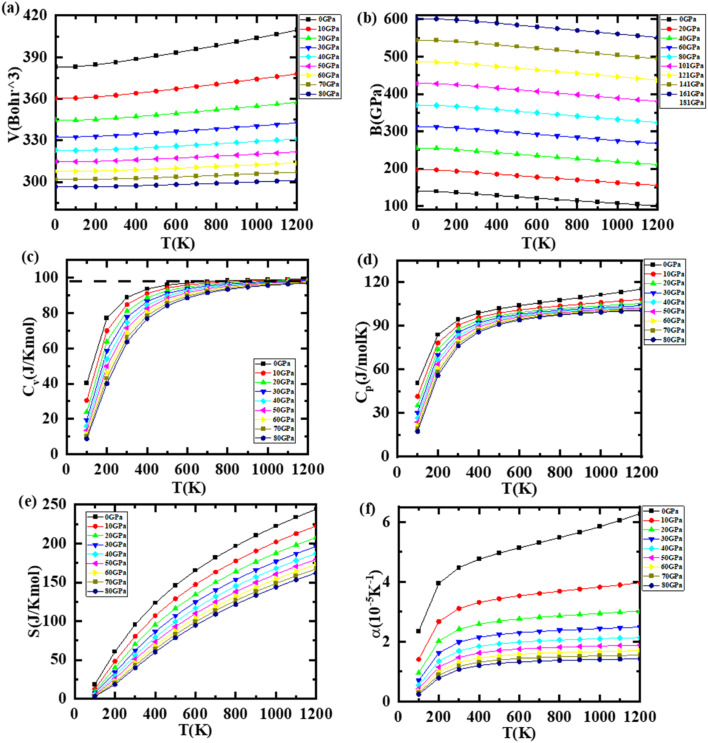
Variation of (a) volume *V*, (b) bulk modulus, (c) specific heat at constant volume *C*_v_, (d) specific heat at constant pressure *C*_p_, (e) entropy *S*, and (f) linear expansion with pressure and temperature for LiZrCoGe.

**Fig. 8:**
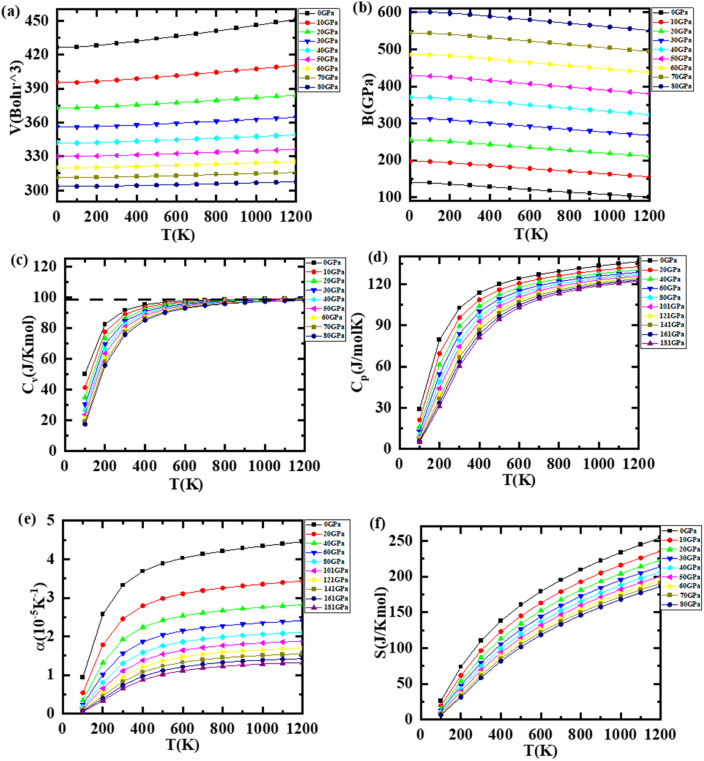
Variation of (a) volume *V*, (b) bulk modulus, (c) specific heat at constant volume *C*_v_, (d) specific heat at constant pressure *C*_p_, (e) entropy *S*, and (f) linear expansion with pressure and temperature for LiZrCoSn.

The thermal as well as pressure response of materials is also very crucial as they play a vital role in their structural, elastic, mechanical, electronic, and vibrational properties. The thermodynamic variables such as specific heat, entropy, Debye temperature, and thermal expansion coefficient were explored using GIBBS software,^28^ which uses quasi-harmonic Debye approximation, and are presented in Fig. 7(a)–(e) and 8(a)–(e) in the case of LiZrCoGe and LiZrCoSn, respectively, in the range up to 1200 K and 80 GPa. As observed from Fig. 7(a) and 8(a), the volume of compounds is found to be increasing with the applied temperature, revealing that they undergo expansion with the temperature. As the pressure increases, the volume gradually decreases for both compounds. The magnitude of the volume of the unit cell of LiZrCoSn is larger compared to that of LiZrCoGe, which might be due to the increased atomic size of the X atom while going from Ge to Sn.^66^ The temperature dependence of the bulk modulus for both compounds is illustrated in Fig. 7(b) and 8(b). As observed in Fig. 7 and 8, the bulk modulus slightly decreases with the temperature whereas a sharp dependence with pressure is observed for both compounds.^67^ The temperature dependence of the heat capacity of both compounds is computed and presented in Fig. 7(c) and 8(c). It is observed that both compounds follow the Dulong–Petit law at higher temperatures. It is seen that *C*_v_ increases rapidly with temperatures up to 800 K, which can be elucidated using Debye's model *C*_v_ ∝ *T*^3^; beyond 800 K, their behavior follows the Dulong–Pettit law *C*_v_ = 3*R* where *R* is the universal gas constant. This rule states that *C*_v_ reaches a constant value of 99.5 J K^−1^ mol^−1^ and 99.98 J K^−1^ mol^−1^ for LiZrCoGe and LiZrCoSn, respectively, after 800 K. However, when the temperature increases, the entropy (*S*), which represents the degree of disorders (see Fig. 7(d) and 8(d)) also increases and decreases with the pressure in both the explored compounds.^68^ The temperature dependence of the Debye temperature *θ*_D_ is also studied for both compounds and illustrated in Fig. 7(e) and 8(e), and it was found to decrease with the temperature but increase with the pressure. The temperature dependence of the thermal expansion coefficient *α* of both compounds has been studied and (see Fig. 7(f) and 8(f)) observed to have a linear increase up to 200 K and gradual increase up to 1200 K at 0 GPa for LiZrCoGe, whereas for LiZrCoSn, after 400 K, for all pressures, it becomes almost constant till 1200 K.

### 3.7. Thermoelectric properties

**Fig. 9:**
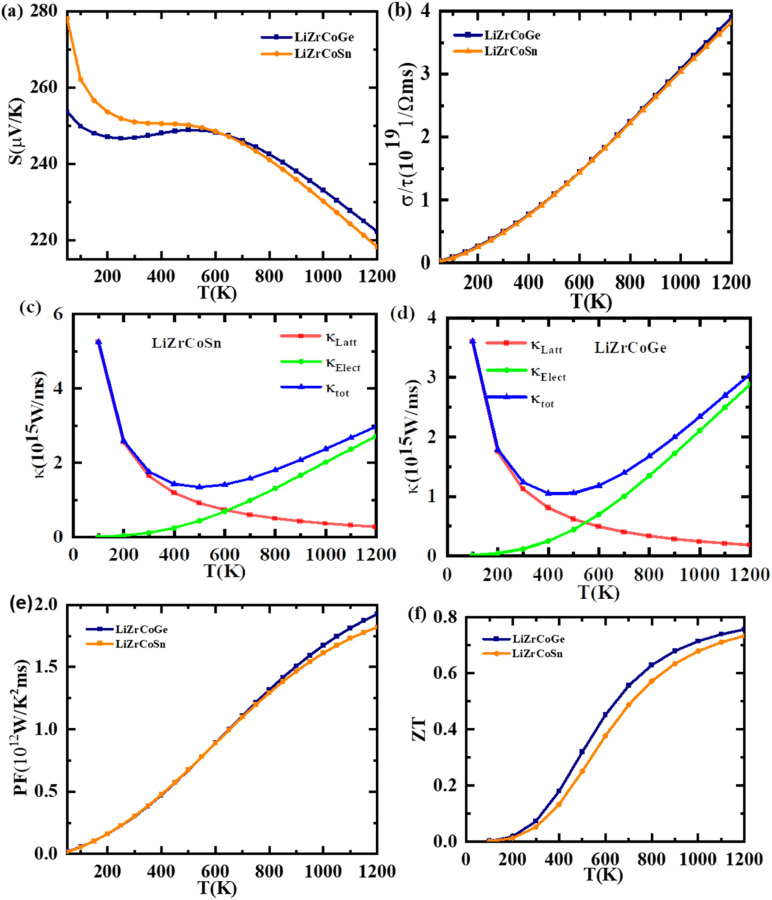
Variation of thermoelectric parameters with temperature for LiZrCoX (X = Ge, Sn).

**Fig. 10:**
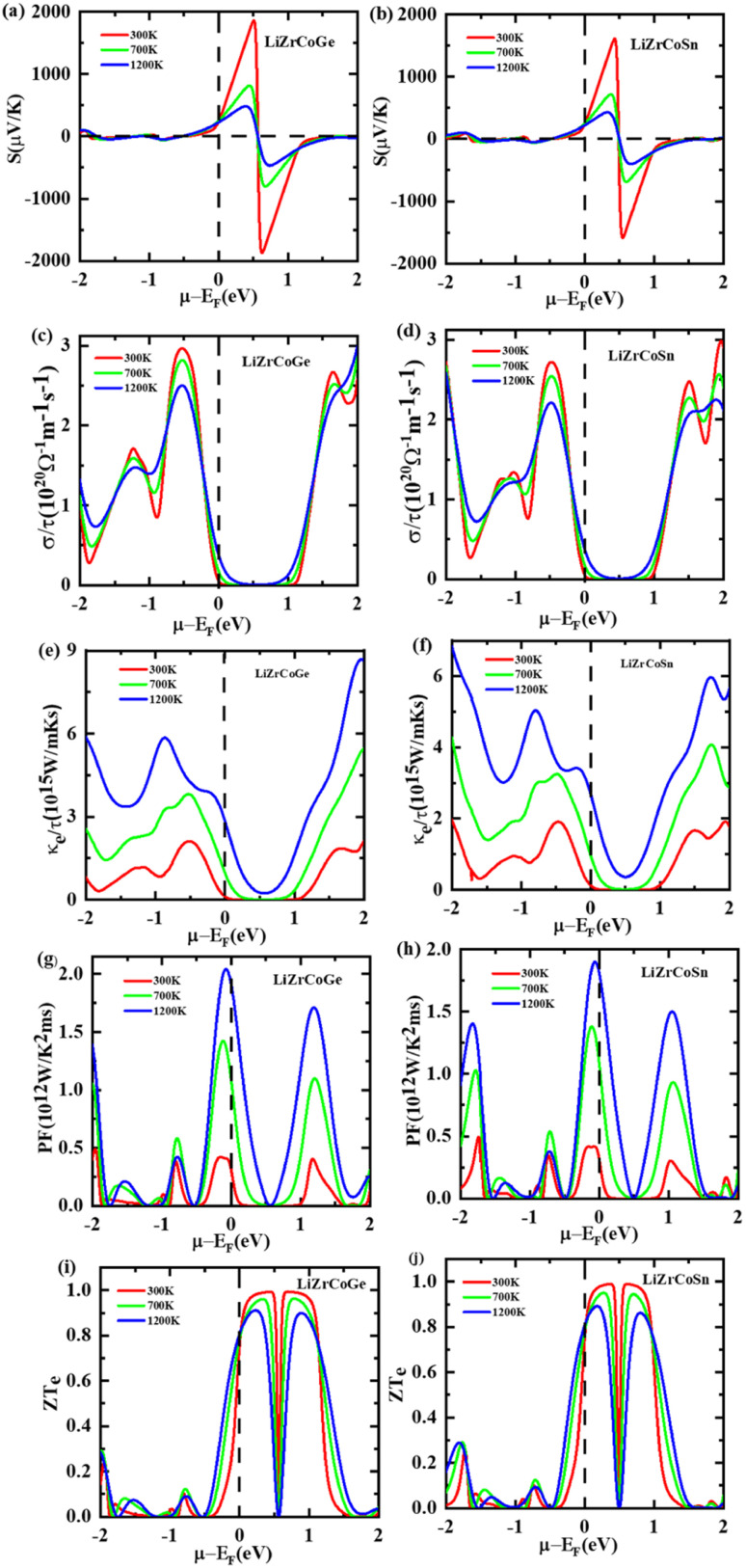
Variation of thermoelectric parameters with chemical potential for LiZrCoX (X = Ge, Sn).

The thermoelectric properties of materials rely on the energy dispersion relations and particularly on the nature of *E*(*K*) at the Fermi level. The comportment of thermoelectric materials can be assessed by their figure of merit (*ZT*). It is used to determine the leeway of using an alloy for thermoelectric applications.^69,70^ The thermoelectric transport properties such as Seebeck coefficient, electrical conductivity, thermal conductivity, and power factor of LiZrCoX (X = Ge, Sn) have been studied with the help of semiclassical Boltzmann theory as implemented in BoltzTraP code.^27^ The temperature and chemical potential dependence of these transport properties of both the investigated compounds have been performed to determine whether they can be assessed as potential candidates for the various thermoelectric applications and illustrated in Fig. 9 and 10. The Seebeck coefficient is one of the thermoelectric parameters that is used to determine the charge carrier responsible for conduction in the thermoelectric transport mechanism. The variation in the Seebeck coefficient with the temperature at the Fermi level has been investigated within the range of 0 to 1200 K and is displayed in Fig. 9(a). It is observed that the value of the Seebeck coefficient at 0 K is 254 μV K^−1^ and 279 μV K^−1^ for LiZrCoGe and LiZrCoSn, respectively, which gets reduced to 247 μV K^−1^ and 251 μV K^−1^ at 300 K, and the same is tabulated in Table 6 with comparison to other reports. The positive value of the obtained Seebeck coefficient indicates the presence of holes as majority charge carriers in both the explored compounds.^71^ The value of the Seebeck coefficient is found to be continuously decreasing up to 1200 K as seen in Fig. 9(a) for both materials.

**Table 6:** Room temperature (300 K) thermoelectric properties of LiZrCoX (X = Ge, Sn)

Compound	*S* (μV K^−1^)	*σ*/*τ* (10^18^ Ω^−1^ m^−1^ s^−1^)	*S* ^2^ *σ*/*τ* (10^11^ W m^−1^ K^−2^ s^−1^)	*κ*_e_/*τ* (10^15^ W m^−1^ K^−1^ s^−1^)
LiZrCoGe	247	4.93	3.01	0.114
LiZrCoSn	251	4.82	3.04	0.119
Other study^78^	20	1.7		1.1

The electrical conductivity with respect to the constant relaxation time, *i.e.*, *σ*/*τ* of these compounds, was also studied up to 1200 K and is presented in Fig. 9(b). The *σ*/*τ* curves are found to be overlapping for both compounds. It is observed that the electrical conductivity of the studied materials is found to be gradually increasing with temperature, which again confirms the semiconducting behavior of these compounds.^72,73^ The electronic thermal conductivity of the studied compounds is also computed and its dependence on temperature is displayed in Fig. 9(c). The total thermal conductivity *κ*_tot_ is the sum of electronic thermal conductivity *κ*_Elec_ and lattice thermal conductivity *κ*_Latt_. The electronic contribution to the total thermal conductivity for both compounds have been computed using BoltzTraP code, and the lattice contribution is computed using Slack equation as 
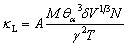
. The variation in *κ*_tot_, *κ*_Elect_, and *κ*_Latt_ with temperature has been studied for both compounds and is displayed in Fig. 9(d). The electronic thermal conductivity for both compounds is found to be linearly increasing with temperatures up to 1200 K, which measures the free electron vibrations in the explored materials, whereas the lattice thermal conductivity decreases sharply with the temperature due to the scattering of phonons.^74^ The total thermal conductivity *κ*_tot_ for both the LiZrCoX compounds is found to be linearly decreasing up to 400 K, after which it increases with temperatures up to 1200 K. Low thermal conductivity and high electrical conductivity are characteristics of good thermoelectric materials. Therefore, the studied LiZrCoGe and LiZrCoSn compounds are good thermoelectric materials up to 400 K. The variation of power factor (PF) with temperature has been studied for both compounds and is displayed in Fig. 9(d). The power factor curves overlap for both compounds and gradually increase with temperature and found to have 1.9 × 10^12^ W K^−2^ m^−1^ s^−1^ and 1.6 × 10^12^ W K^−2^ m^−1^ s^−1^ for LiZrCoGe and LiZrCoSn, respectively. The figure of merit (*ZT*) is one of the crucial metrics to determine whether the material is a good thermoelectric or not. Its dimensionless relation is given as 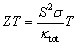. The calculated figure of merit is found to gradually increase with the temperature and attains a maximum value of 0.78 and 0.76 for LiZrCoGe and LiZrCoSn, respectively, as presented in Table 6, with comparison to available reports. The chemical potential dependence on the Seebeck coefficient has been studied and illustrated in Fig. 10(a). The doping range of material is represented as positive for n-type and negative for p-type along the chemical potential axis (*μ*–*ε*_F_). It can be seen from Fig. 10(a) and (b) that Seebeck coefficients are found to be decreasing with the temperature for both compounds.^75,76^ The maximum value of the Seebeck coefficient is found to be 1821 μV K^−1^ for LiZrCoGe at 300 K and 0.5 eV compared to that of 1580 μV K^−1^ for LiZrCoSn. The magnitude of the Seebeck coefficient is found to be reduced as we go from LiZrCoGe to LiZrCoSn, which might be due to the diminishing nature of the energy band gap of the compounds.^77–79^ The electrical conductivity per relaxation time (*σ*/*τ*) of both compounds are studied in the chemical potential ranging from −2.0 eV to 2.0 eV at different temperatures such as 300 K, 700 K, and 1200 K and are demonstrated in Fig. 10(c) and (d). The value of *σ*/*τ* is found to be zero in the chemical range from 0.0 eV to 1.0 eV, after which it starts to increase with both sides of chemical potential and is observed to have opposite behavior above ±1.5 eV for LiZrCoGe in the studied chemical potential range. A similar trend is followed for LiZrCoSn and was observed to have opposite behavior of increasing with the temperature above ±1.3 eV. The maximum value of *σ*/*τ* was found to be at the negative side of the chemical potential *μ*–*ε*_F_, *i.e.*, for p-type in both compounds.

The variation of electronic thermal conductivity (*κ*_e_/*τ*) with the chemical potential is also studied and presented in Fig. 10(e) and (f). *κ*_e_/*τ* is found to be zero in the chemical potential range from 0.3 eV to 0.7 eV. The electronic thermal conductivity of both compounds is found to be increasing with temperature as we go from 300 K to 1200 K. The chemical potential dependence of the power factor (PF) has been studied for both compounds in the chemical potential range from −2.0 eV to 2.0 eV at 300 K, 700 K, and 1200 K, respectively, and is illustrated in Fig. 10(g) and (h). LiZrCoGe possesses a peak value of power factor of 2.2 × 10^12^ W K^−2^ m^−1^ s^−1^ at −0.2 eV whereas LiZrCoSn has an optimal value of 1.8 × 10^12^ W K^−2^ m^−1^ s^−1^ at −0.2 eV. The variation of figure of merit (*ZT*_e_) with the chemical potential has been studied in the chemical potential range of −2.0 eV to 2.0 eV and is presented in Fig. 10(i) and (j) at different temperatures of 300 K, 700 K, and 1200 K, respectively. It can be seen from Fig. 10(i) that both compounds attain *ZT*_e_ = 0.8 at room temperature in the vicinity of the Fermi level and reaches an optimal value of approximately unity at 0.3 eV. In the p-type region, *ZT*_e_ increases with the temperature whereas in the range of 0 eV to 1.3 eV in the n-type region, *ZT*_e_ decreases with the temperature in the case of LiZrCoGe and LiZrCoSn.

## 4. Conclusion

With the aid of DFT as implemented in the WIEN2k code, the structural, magnetic, electrical, elastic, vibrational, optical, thermodynamic, and thermoelectric properties of quaternary LiZrCoX (X = Ge, Sn) compounds are thoroughly examined. The computed equilibrium structural data are in good agreement with the prevailing similar theoretical and experimental literature. The studied compounds are found to be stable in Type-3 crystal structure as seen from the volume optimization curves. The tabulated cohesive energy, formation energy, and phonon band spectrum of these compounds depict their chemical, thermodynamic, as well as dynamic stability in the cubic phase. The computed second-order elastic constants of LiZrCoX compounds satisfy the mechanical stability of these compounds. The variation in the optical properties such as dielectric function, refractive index, extinction coefficient, absorption coefficient, reflectivity, energy loss function, and optical conductivity with the incident electromagnetic radiations up to 12 eV are also studied. As an outcome, LiZrCoX compounds have the potential to be novel and intriguing thermoelectric materials that can also be used in optoelectronic and energy harvesting systems.

## Data availability

Data will be available on reasonable request to the corresponding author.

The raw/processed data required to reproduce these findings cannot be shared at this time due to the ongoing research on this.

## Conflicts of interest

The authors declare that they have no known competing financial interests or personal relationships that could have appeared to influence the work reported in this paper.

## Supplementary Material

## References

[cit1] Liu Z., Guo S., Wu Y., Mao J., Zhu Q., Zhu H., Pei Y., Sui J., Zhang Y., Ren Z. (2019). Adv. Funct. Mater..

[cit2] Wolf M., Hinterding R., Feldhoff A. (2019). Entropy.

[cit3] Dehkordi A. M., Zebarjadi M., He J., Tritt T. M. (2015). Mater. Sci. Eng., R.

[cit4] Manzoor M., Behera D., Sharma R., Iqbal M. W., Mukherjee S. K., Khenata R., Bin-Omran S., Alshahrani T., El Shiekh E., Ouahrani T. (2023). J. Solid State Chem..

[cit5] Yang J., Meisner G. P., Chen L. (2004). Appl. Phys. Lett..

[cit6] Liu Z., Sun J., Mao J., Zhu H., Ren W., Zhou J., Wang Z., Singh D. J., Sui J., Chu C.-W. (2018). Proc. Natl. Acad. Sci. U. S. A..

[cit7] Behera D., Al-Qaisi S., Manzoor M., Sharma R., Srivastava V., mana Al-Anazy M., El Shiekh E., Mukherjee S. K. (2023). Mater. Sci. Eng., B.

[cit8] Behera D., Mukherjee S. K. (2023). JETP Lett..

[cit9] Zhang C., Huang H., Wu C., Zhu Z., He Z., Liu G. (2020). Front. Phys..

[cit10] Bainsla L., Mallick A. I., Raja M. M., Nigam A. K., Varaprasad B. S. D. C. S., Takahashi Y. K., Alam A., Suresh K. G., Hono K. (2015). Phys. Rev. B: Condens. Matter Mater. Phys..

[cit11] Bahramian S., Ahmadian F. (2017). J. Magn. Magn. Mater..

[cit12] Zhang Y. J., Liu Z. H., Li G. T., Ma X. Q., Liu G. D. (2014). J. Alloys Compd..

[cit13] Idrissi S., Ziti S., Labrim H., Bahmad L. (2021). Chin. J. Phys..

[cit14] Kaur T., Singh J., Goyal M., Kaur K., Khandy S. A., Bhat M. A., Sharopov U. B., Dhiman S., Wani A. F., Rani B. (2022). Phys. Scr..

[cit15] Haleoot R., Hamad B. (2019). J. Phys.: Condens. Matter.

[cit16] Kara H., Kahaly M. U., Özdoğan K. (2018). J. Alloys Compd..

[cit17] Idrissi S., Labrim H., Ziti S., Bahmad L. (2020). J. Supercond. Novel Magn..

[cit18] Abraham J. A., Sharma R., Dar S. A., Chowdhury S. (2022). Int. J. Energy Res..

[cit19] Gupta Y., Sinha M. M., Verma S. S. (2021). J. Solid State Chem..

[cit20] Singh J., Kaur K., Khandy S. A., Goyal M., Dhiman S., Verma S. S. (2022). Mater. Today:
Proc..

[cit21] Singh J., Kaur K., Khandy S. A., Dhiman S., Goyal M., Verma S. S. (2021). Int. J. Energy Res..

[cit22] Blaha P., Schwarz K., Sorantin P., Trickey S. B. (1990). Comput. Phys. Commun..

[cit23] Schwarz K., Blaha P., Madsen G. K. H. (2002). Comput. Phys. Commun..

[cit24] Blaha P. , Schwarz K., Madsen G., Kvasnicka D. and Luitz J., Materials Chemistry, TU Vienna, http://www.wien2k.at

[cit25] Monkhorst H. J., Pack J. D. (1976). Phys. Rev. B: Solid State.

[cit26] Jamal M., Bilal M., Ahmad I., Jalali-Asadabadi S. (2018). J. Alloys Compd..

[cit27] Madsen G. K. H., Singh D. J. (2006). Comput. Phys. Commun..

[cit28] Otero-de-la-Roza A., Luaña V. (2011). Comput. Phys. Commun..

[cit29] Hafner J. (2008). J. Comput. Chem..

[cit30] Graf T., Casper F., Winterlik J., Balke B., Fecher G. H., Felser C. (2009). Z. Anorg. Allg. Chem..

[cit31] Alijani V., Ouardi S., Fecher G. H., Winterlik J., Naghavi S. S., Kozina X., Stryganyuk G., Felser C., Ikenaga E., Yamashita Y. (2011). Phys. Rev. B: Condens. Matter Mater. Phys..

[cit32] Wang X., Cheng Z., Liu G., Dai X., Khenata R., Wang L., Bouhemadou A. (2017). IUCrJ.

[cit33] Huang H.-L., Tung J.-C., Jeng H.-T. (2021). Phys. Chem. Chem. Phys..

[cit34] Katsura T., Tange Y. (2019). Minerals.

[cit35] Meng F., Hao H., Ma Y., Guo X., Luo H. (2017). J. Alloys Compd..

[cit36] Zhao J.-S., Gao Q., Li L., Xie H.-H., Hu X.-R., Xu C.-L., Deng J.-B. (2017). Intermetallics.

[cit37] Manzoor M., Behera D., Sharma R., Iqbal M. W., Mukherjee S. K. (2023). Mater. Sci. Eng., B.

[cit38] Gao S., Broux T., Fujii S., Tassel C., Yamamoto K., Xiao Y., Oikawa I., Takamura H., Ubukata H., Watanabe Y. (2021). Nat. Commun..

[cit39] Singh J., Kaur T., Goyal M., Kaur K., Verma S. S., Sinha M. M. (2023). Mater. Today: Proc..

[cit40] Miri M., Ziat Y., Belkhanchi H., Zarhri Z., El Kadi Y. A. (2023). Phys. B.

[cit41] Raïâ M. Y., Masrour R., Hamedoun M., Kharbach J., Rezzouk A., Hourmatallah A., Benzakour N., Bouslykhane K. (2023). Opt. Quantum Electron..

[cit42] Katubi K. M., Zafar M., Tufail S. F., Shakil M., Ahmed A., Alrowaili Z. A., Al-Buriahi M. S. (2023). Phys. B.

[cit43] Özdoğan K., Şaşıoğlu E., Galanakis I. (2013). J. Appl. Phys..

[cit44] Amari D., Mokhtari M., Dahmane F., Benabdellah G. (2023). Emergent Mater..

[cit45] Tindibale E., Mulwa W. M., Adetunji B. I. (2023). Phys. B.

[cit46] Waller I. (1956). Acta Crystallogr..

[cit47] Bouhemadou A., Khenata R. (2007). Phys. Lett. A.

[cit48] Bounab S., Bentabet A. (2023). Indian J. Phys..

[cit49] Kube C. M. (2016). AIP Adv..

[cit50] Fatima B., Chouhan S. S., Acharya N., Sanyal S. P. (2014). Intermetallics.

[cit51] Haines J., Leger J. M., Bocquillon G. (2001). Annu. Rev. Mater. Res..

[cit52] Teli N. A., Sirajuddeen M. M. S. (2020). Phys. Lett. A.

[cit53] Pugh S. F. (1954). London, Edinburgh Dublin Philos. Mag. J. Sci..

[cit54] Pugh S. F. (1954). London, Edinburgh Dublin Philos. Mag. J. Sci..

[cit55] Eberhart M. E., Jones T. E. (2012). Phys. Rev. B: Condens. Matter Mater. Phys..

[cit56] Behera D., Mohammed B., Taieb S., Mokhtar B., Al-Qaisi S., Mukherjee S. K. (2023). Eur. Phys. J. Plus.

[cit57] Manzoor M., Behera D., Sharma R., Iqbal M. W., Mukherjee S. K., Khenata R., Alarfaji S. S., Alzahrani H. A. (2023). Mater. Today Commun..

[cit58] Kuzmenko A. B. (2005). Rev. Sci. Instrum..

[cit59] Behera D., Manzoor M., Maharana M., Iqbal M. W., Zahid T., Lakra S., Mukherjee S. K., Alarfaji S. S. (2023). Phys. B.

[cit60] Azam A., Sharma R., Behera D., Raza H. H., Ali H. S., Abdelmohsen S. A. M., Abdelbacki A. M. M., Mukherjee S. K. (2023). RSC Adv..

[cit61] Kramers H. A. , in Atti Cong. Intern. Fisica (Transactions of Volta Centenary Congress) Como, 1927, vol. 2, pp. 545–557

[cit62] Seddik T., Behera D., Batouche M., Ouerghui W., Ben Abdallah H., Sarkar R. K., Salah M. M., Shaker A., Mukherjee S. K. (2023). Crystals.

[cit63] Behera D., Sharma R., Ullah H., Waheed H. S., Mukherjee S. K. (2022). J. Solid State Chem..

[cit64] Behera D., Mukherjee S. K. (2023). Mater. Sci. Eng., B.

[cit65] Behera D. , Manzoor M., Iqbal M. W., Lakra S. and Mukherjee S. K., Optical, and Thermoelectric Behavior of EU Based EuAg_2_Y_2_ (Y = S/Se): For Solar Cell Applications

[cit66] Behera D., Dixit A., Kumari K., Srivastava A., Sharma R., Mukherjee S. K., Khenata R., Boumaza A., Bin-Omran S. (2022). Eur. Phys. J. Plus.

[cit67] Behera D., Dixit A., Nahak B., Srivastava A., Dubey S., Sharma R., Mishra A. K., Mukherjee S. K. (2022). Phys. Lett. A.

[cit68] Manzoor M., Behera D., Chowdhury S., Sharma R., Iqbal M. W., Mukherjee S. K., Alarfaji S. S., Alzahrani H. A. (2022). Comput. Theor. Chem..

[cit69] Abraham J. A., Behera D., Kumari K., Srivastava A., Sharma R., Mukherjee S. K. (2022). Chem. Phys. Lett..

[cit70] Behera D., Mukherjee S. K. (2022). JETP Lett..

[cit71] Saxena A., Dixit A., Behera D., Abraham J. A., Sharma R., Mukherjee S. K. Mater. Today: Proc..

[cit72] Behera D., Manzoor M., Mukherjee S. K. (2022). Comput. Condens. Matter.

[cit73] Manzoor M., Bahera D., Sharma R., Tufail F., Iqbal M. W., Mukerjee S. K. Int. J. Energy Res..

[cit74] Behera D., Mukherjee S. K. (2022). Chemistry.

[cit75] Behera D., Dixit A., Nahak B., Srivastava A., Sharma R., Khenata R., Bin-Omran S., Abdelmohsen S. A. M., Abdelbacki A. M. M., Mukherjee S. K. (2023). Mater. Today Commun..

[cit76] Satyam J. K., Saini S. M. (2023). J. Comput. Chem..

[cit77] Guo D., Li C., Li K., Shao B., Chen D., Ma Y., Sun J., Cao X., Zeng W., Yang R. (2021). Phys. E.

[cit78] Hossain M. A., Rahman M. T., Khatun M., Haque E. (2018). Comput. Condens. Matter.

[cit79] Baaziz H., Ghellab T., Güler E., Charifi Z., Uğur Ş., Güler M., Uğur G. (2022). J. Supercond. Novel Magn..

